# Colour break in reverse bicolour daffodils is associated with the presence of *Narcissus mosaic virus*

**DOI:** 10.1186/1743-422X-8-412

**Published:** 2011-08-21

**Authors:** Donald A Hunter, John D Fletcher, Kevin M Davies, Huaibi Zhang

**Affiliations:** 1 The New Zealand Institute for Plant & Food Research Limited, Private Bag 11600, Palmerston North, New Zealand; 2 The New Zealand Institute for Plant & Food Research Limited, Private Bag 4704, Christchurch, New Zealand

## Abstract

**Background:**

Daffodils (*Narcissus pseudonarcissus*) are one of the world's most popular ornamentals. They also provide a scientific model for studying the carotenoid pigments responsible for their yellow and orange flower colours. In reverse bicolour daffodils, the yellow flower trumpet fades to white with age. The flowers of this type of daffodil are particularly prone to colour break whereby, upon opening, the yellow colour of the perianth is observed to be 'broken' into patches of white. This colour break symptom is characteristic of potyviral infections in other ornamentals such as tulips whose colour break is due to alterations in the presence of anthocyanins. However, reverse bicolour flowers displaying colour break show no other virus-like symptoms such as leaf mottling or plant stunting, leading some to argue that the carotenoid-based colour breaking in reverse bicolour flowers may not be caused by virus infection.

**Results:**

Although potyviruses have been reported to cause colour break in other flower species, enzyme-linked-immunoassays with an antibody specific to the potyviral family showed that potyviruses were not responsible for the occurrence of colour break in reverse bicolour daffodils. Colour break in this type of daffodil was clearly associated with the presence of large quantities of rod-shaped viral particles of lengths 502-580 nm in tepals. Sap from flowers displaying colour break caused red necrotic lesions on *Gomphrena globosa*, suggesting the presence of potexvirus. Red necrotic lesions were not observed in this indicator plant when sap from reverse bicolour flowers not showing colour break was used. The reverse transcriptase polymerase reactions using degenerate primers to carla-, potex- and poty-viruses linked viral RNA with colour break and sequencing of the amplified products indicated that the potexvirus *Narcissisus mosaic virus *was the predominant virus associated with the occurrence of the colour break.

**Conclusions:**

High viral counts were associated with the reverse bicolour daffodil flowers that were displaying colour break but otherwise showed no other symptoms of infection. *Narcissus mosaic virus *was the virus that was clearly linked to the carotenoid-based colour break.

## Background

Colour breaking of flowers is a long-documented type of variegation in the flower where the usual pigment pattern of the perianth is changed to irregular patches or streaks of pigmentation. Colour break occurs in many plant species including camellia [1], orchids [2], lilies and tulips [3]. In these flowers the colour break is due to changes in the concentrations of vacuole-localised anthocyanin pigments in the upper epidermal layer of the tepals. In tulips these changes have been termed 'full break' where colour is removed in tepal areas, 'self break' where it is intensified, and 'average break' where full and self break occur simultaneously [4]. Colour breaking in tulips was reported as far back as 1576 when Clusius described a variegation of the flower colour and a general weakening of the plants leading to loss of varieties [5]. Because of their beauty and rarity these tulip flowers were highly sought after and this resulted in a phenomenon in the Netherlands known as tulipomania where between 1634 and 1637 extraordinarily high prices were paid by financial speculators for the colour-broken tulips [5].

Colour break in tulips is unpredictable and its cause was not known in the seventeenth century, although grafting experiments in 1637 demonstrated that the trait could be transmitted from bulbs of variegated tulips to bulbs of uniformly coloured tulips [5]. It took until 1919 for viruses to be suggested as the cause of the colour break and a further 8 years for sap transmission to be demonstrated [3]. Five distinct potyviruses have been shown to cause colour break in tulips [6]. The infected flowers are now considered undesirable as the viral infection often leads to reduced health and vigour leading to economic losses for the ornamental industry.

Daffodil (*Narcissus pseudonarcissus*) flowers also colour break. In daffodil, the colour break is due to changes in the levels of lipid-soluble pigments called carotenoids which, in contrast to anthocyanins, accumulate in the chromoplasts of cells. In most varieties of *N. pseudonarcissus *colour break is rare, usually occurring only in plants displaying leaf damage and greatly reduced vigour. Viruses are likely the cause of the colour break in these flowers. For example, 74% of 'Minister Talma' daffodils naturally infected with *Narcissus yellow stripe *(NYSV) and *Tobacco rattle virus *(TRV) were found to have white blotches on their perianths [7] and *Narcissus late season yellows virus *(NLSYV), *Narcissus degeneration virus *(NDV) and *Narcissus symptomless virus *(NSV) were found in two daffodils showing flower distortion, flower colour break and leaf mottling [8]

The rarity of colour break in *N. Pseudonarcissus *flowers is curious given the prevalence of viruses in the species and that the viruses are often present in complexes of up to four different types [9,10]. Clark and Guy [10] surveyed *Narcissus *spp. for viral infection in the Otago Province of New Zealand using the enzyme-linked-immunosorbent assay (ELISA) and mechanical transmission tests and detected *Narcissus mosaic virus *(NMV), *Cucumber mosaic virus *(CMV), *Narcissus latent virus *(NLV), *Narcissus tip necrosis virus *(NTNV) and *Narcissus yellow stripe virus *(NYSV) at high incidence in all five sites examined. Many of these viruses were present in the daffodils as mixed infections, yet the researchers reported that they did not observe colour break. Similarly, tepals of 'Dutch Master' flowers that were infected with at least three different types of viruses (NLSYV, NLV and NYSV) showed no signs of colour break (D. A. Hunter *unpublished data*).

Reverse bicolour flowers are a particular type of *N. pseudonarcissus *prone to colour break. This type of flower was first noted in the 1920s and is characterised by opening yellow but having its corona fade to white with age. In contrast with other daffodils, the colour breaking of the reverse bicolour flower occurs in what appear to be otherwise healthy plants. Because of this, debate remains as to whether viruses are the cause. In this study we have investigated whether viral infection is linked to the carotenoid-based colour break in reverse bicolour daffodil tepals.

## Results

### Colour break in daffodils

**Figure 1:**
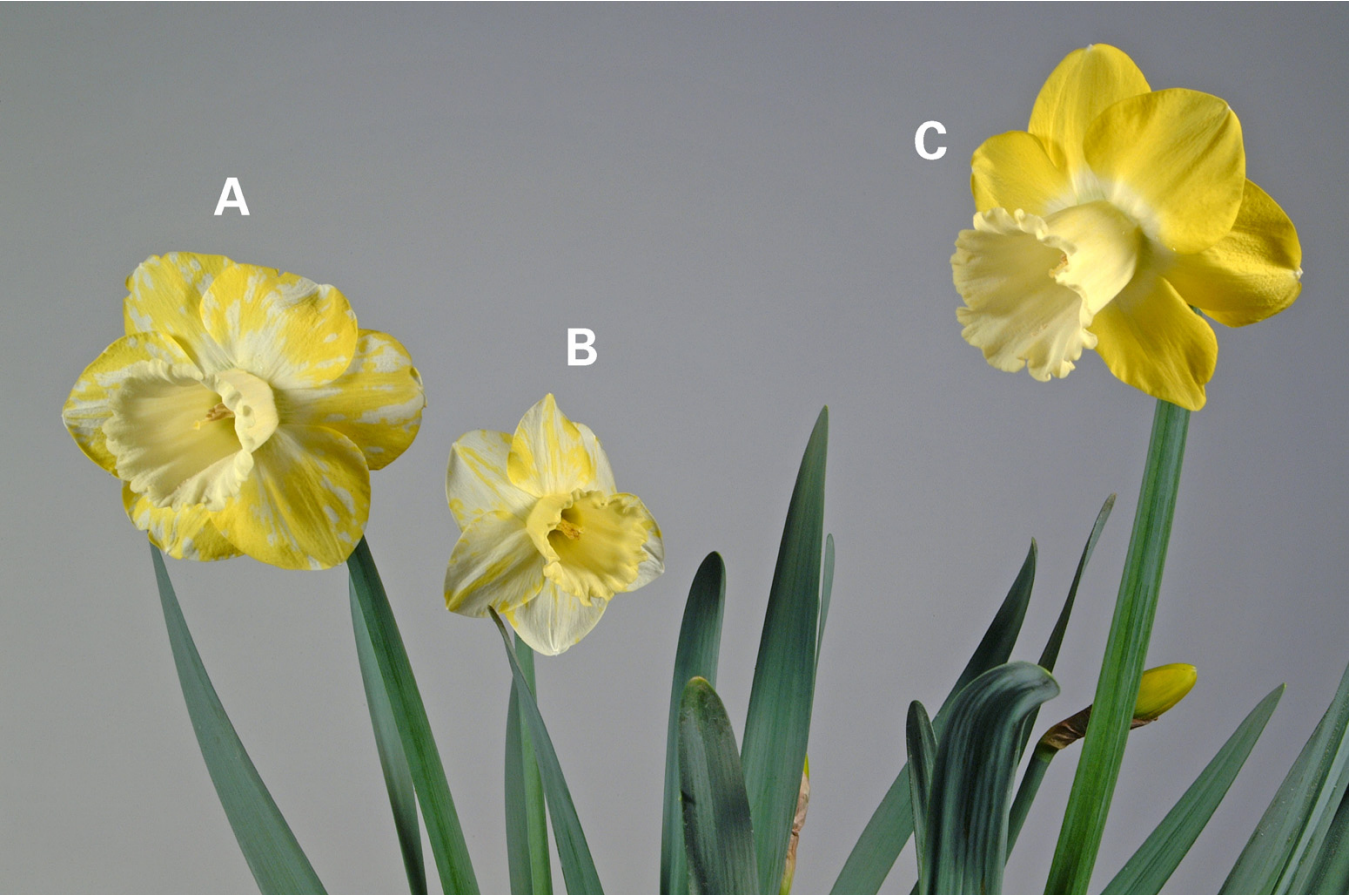
**Colour break symptoms**. (A, B) 'Lighthouse Reef' flowers showing symptoms of colour break. (C) Normal appearance of flower.

The occurrence of colour break was rare at the sampling sites examined and most common in the reverse bicolour type. Colour break was characterised by the presence of irregular white patches where normally yellow pigment would have been present (Figure 1). Colour break could be observed while the flower was still in bud.

### Tepal extracts from colour-broken flowers caused symptoms on *Gomphrena globosa* plants

**Figure 2:**
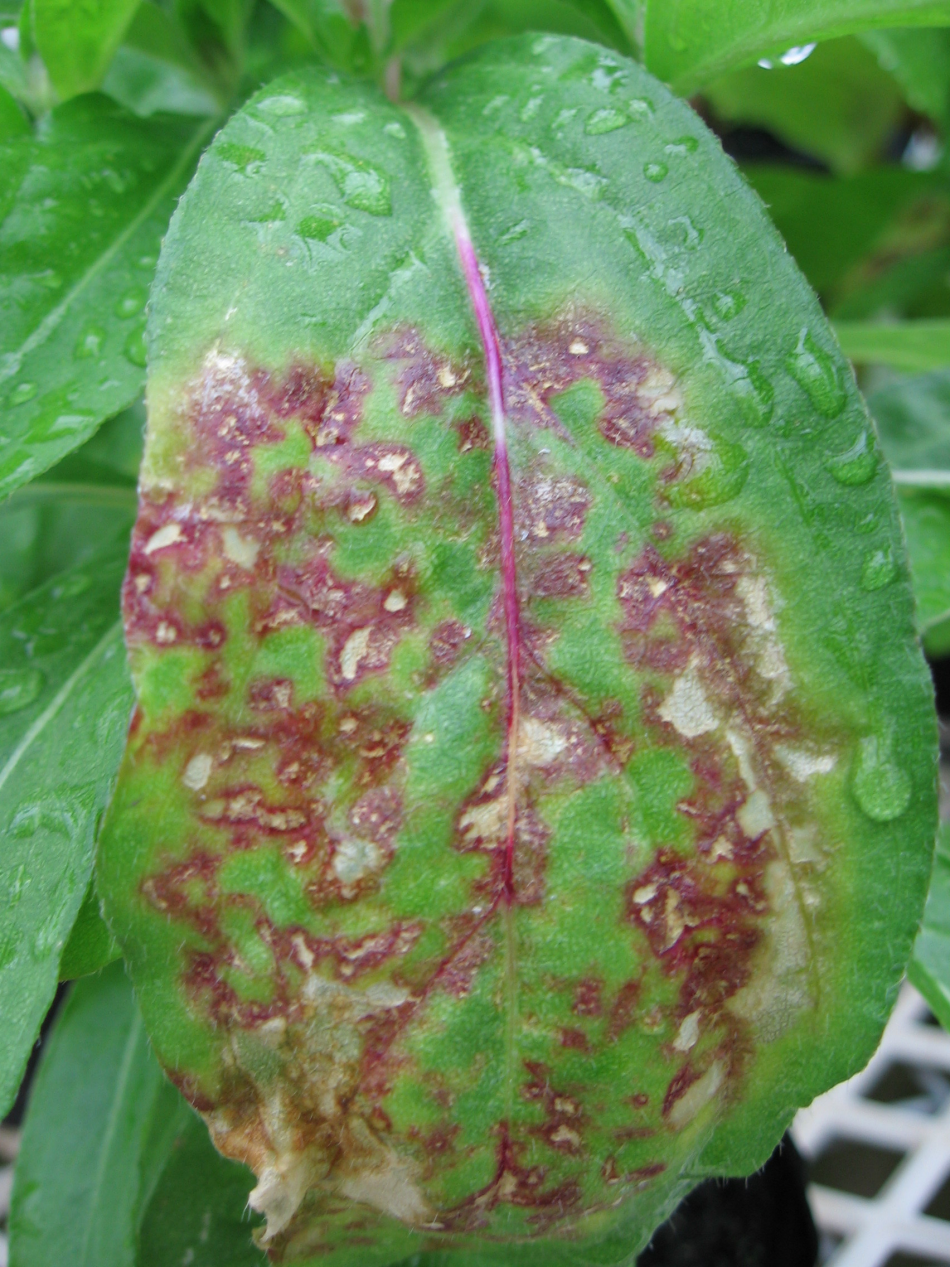
**Red ringspot lesions appeared in *Gomphrena globosa *after inoculation with tepal extracts from colour-broken flowers**.

Indicator plants were initially used to test for viral presence in 10 colour-broken reverse-bicolour flowers collected from three different locations in New Zealand (Nelson, Ashhurst, and Foxton). Tepal extracts from all 10 colour-broken flowers caused formation of red ringspot lesions in *G. globosa *leaves 10 days post inoculation (Figure 2, Table 1). By contrast, no lesions were observed in *G. globosa *leaves when the tepal extracts from non-colour-broken flowers were used as the inocula indicating that colour break correlated with the presence of a particular virus(s). Local lesions on *Nicotiana tabacum *and *Chenopodium amaranticolor *were also observed after their inoculation with extracts from colour-broken flowers of 'Twilight Zone' and 'Twelve Gauge' respectively, however symptoms on *G. globosa *presented the most consistent relationship between the presence and absence of colour break on an indicator host.

**Table 1:** Lesion formation occurrence in indicator plants following inoculation with extracts from colour-broken and non-colour-broken *Narcissus *flowers

Cultivar name	Location	Colour broken	Host reaction	Potyvirus DIBA-ELISA results
Twilight Zone	Ashhurst	Yes	LL on Gg turning systemic	-
		No	NS	(+)
Twilight Zone	Ashhurst	Yes	LL on Gg turning systemic LL on Nt	(+)
		No	NS	-
Twelve Gauge	Foxton	Yes	LL on Gg turning systemic LL Ca	-
		No	NS	-
Daydream	Nelson	Yes	LL on Gg turning systemic	-
		No	NS	-
Startracker	Ashhurst	Yes	LL on Gg turning systemic	-
		No	NS	-
Honeybird	Foxton	Yes	LL on Gg turning systemic	-
		No	NS	(+)
82/90 (Rich Reward × [Daydream × Empress of Ireland])	Nelson	Yes	LL on Gg turning systemic	-
		No	NS	-
20/87 (Royalist × [1W P Sdg] × Fintona) × Kabonova	Nelson	Yes	LL on Gg turning systemic	-
Daydream Mitsch	Nelson	Yes	LL on Gg turning systemic	(+)
Lighthouse Reef	Ashhurst	Yes	LL on Gg turning systemic	-
Trumpet Warrior	Nelson	No	NS	-
Gold Convention (non reverse bicolor)	Ashhurst	No	NS	+
Control PVY			-	+
Control sap			-	-
LL C am. Twelve Gauge				-
LL G g Daydream				-

Indicator plants *Chenopodium amaranticolor *(Ca), *Gomphrena globosa *(Gg), *Phaseolus vulgaris *(Pv) and *Nicotiana tabacum *(Nt) were inoculated with tepal tissue extracts and the host reaction determined. NS = no symptoms; LL = local lesion. Presence of potyviruses in the tepal extracts was determined by DIBA-ELISA. All daffodil tepals were tested. '+' = positive, '(+)' = weak positive reaction, '-' = negative

### Tepal colour break did not correlate with the presence of potyviruses

Dot immunobinding assay-enzyme linked immunosorbent assay (DIBA-ELISA) using a broad-spectrum anti-potyviral monoclonal antibody showed that there was no correlation between the presence of potyviruses in the tepals and colour break (Table 1). Potyviral proteins were detected in only two out of the 10 colour-broken flowers and were also detected in two flowers not displaying colour break.

### Colour break was associated with the presence of rod-shaped viral particles in tepals

**Figure 3:**
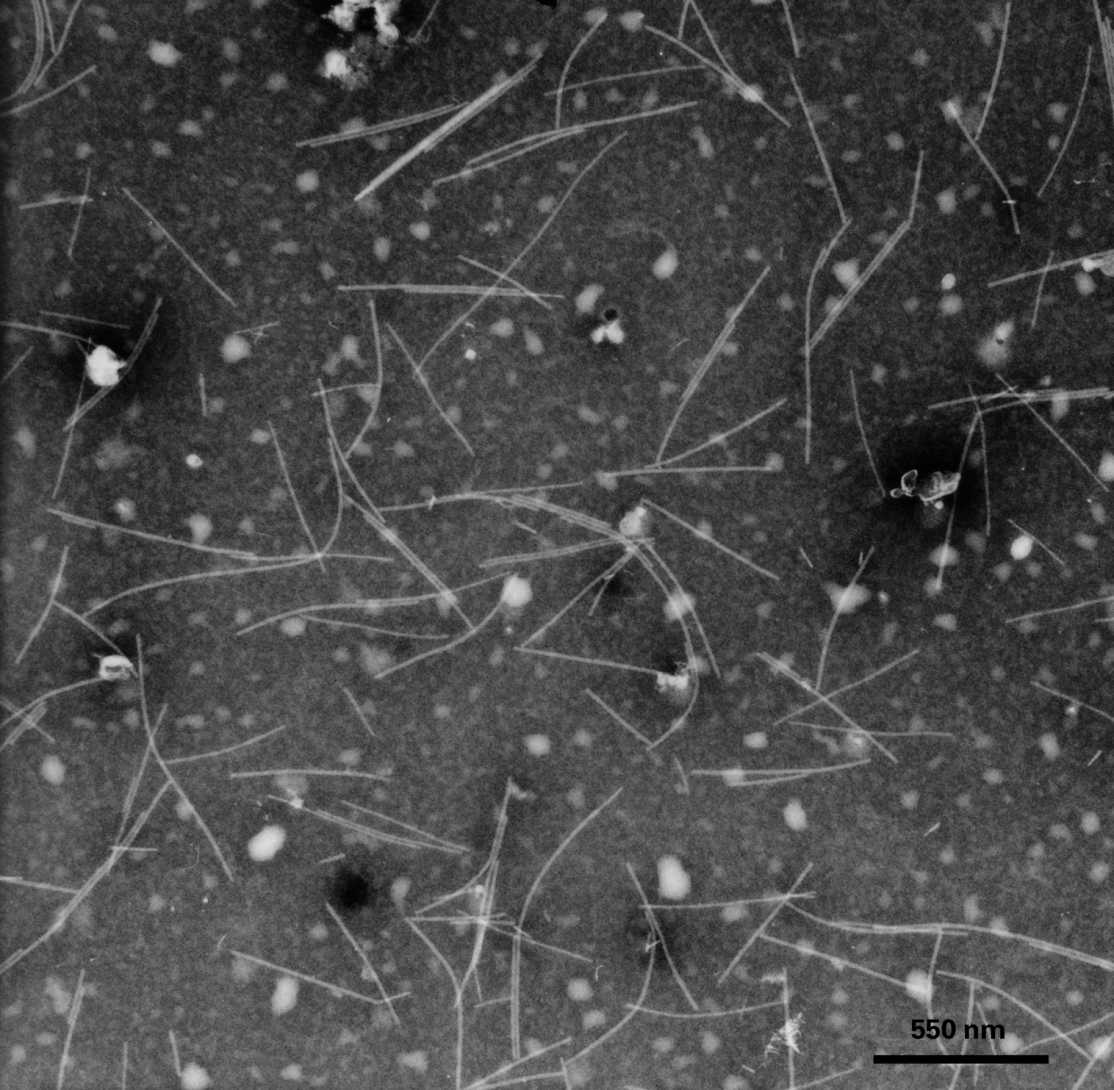
**Electron micrograph showing viral particles present in tepal extracts of colour-broken 'Lighthouse Reef' flowers**.

Transmission electron microscopy (TEM) was used to examine the quantity and morphological characteristics of the viral particles present in a selection of the colour-broken and non-colour-broken flowers used in the indicator plant analysis. The selection encompassed all three geographic locations. TEM revealed that the tepals of all six flowers displaying colour break contained many (> 12 viral particle counts per view of randomly chosen area of TEM grid) rod-shaped viral particles of length 502-580 nm (Figure 3, Table 2). Their shape and length was close to that previously reported by Brunt (1966) for NMV. By contrast, viral particles were either not present, or occurred very rarely (e.g., one viral particle in whole sap extract examined) in the tepals of all three flowers not showing colour break (Table 2).

**Table 2:** Viral particle count in *Narcissus *flowers, leaves and bulbs after electron microscopic examination

Daffodil cultivar	Viral length (nm)	Colour break	Location	No. virus particles tepals	No. virus particles leaves	No. virus particles bulbs
Twilight Zone	580 ± 13	Yes	Ashhurst	29 ± 4	-	-
Twilight Zone	658	No	Ashhurst	0^1^	-	-
Twelve Gauge	543 ± 8	Yes	Foxton	12 ± 3	-	-
Twelve Gauge	637	No	Foxton	0^2^	-	-
21/92A seedling	569 ± 12	Yes	Nelson	51 ± 14	4 ± 1	1 ± 0^2^
21/92A seedling		No	Nelson	0^3^	0^3^	0^3^
Lighthouse Reef	502 ± 14	Yes	Ashhurst	28 ± 5	-	-
Daydream	519 ± 33	Yes	Nelson	20 ± 2	-	-
20/87H seedling	544 ± 3	Yes	Nelson	17 ± 1	-	-
Culfind (offset Bulb)^4^	644 ± 10	Yes	Nelson	15 ± 1	-	-
Culfind (parent bulb)		No	Nelson	2 ± 0	-	-

Viral counts were determined from TEM photos. The photos were taken from at least three randomly chosen observations of a grid under magnification of 21200 X. When randomly chosen grid views did not reveal presence of particles the whole extract was screened to determine presence of viral particles. ^1^Only one virus particle found in whole extract; ^2^Very hard to find virus particles, with extensive screening of petal extract required (i.e., many randomly chosen observations contained no viral particles); ^3^No virus particles found anywhere in extract; ^4^Non reverse bicolour '-' = not measured.

Viral counts were also measured in the leaf and bulb extracts of seedling 21/92A, whose colour-broken tepals had the highest viral counts. In 21/92A, viral counts were found to be much lower in the vegetative tissues compared with the tepal tissues (Table 2).

TEM was also used to examine the presence of viral particles in a colour broken non-reverse-bicolour flower 'Culfind' and its non-colour-broken flower control. In this instance bulbs giving rise to the flowers were conjoined; the non-colour broken flower arose from the mother bulb, whereas the colour-broken flower arose from the attached offset bulb. TEM analysis again revealed that the colour break was associated with higher counts of rod-shaped viral particles. However, interestingly, in this instance, the length of the particles was ~644 nm which was longer than seen in the reverse bicolour tepal extracts (Table 2).

### Colour break correlated with the presence of NMV RNA

**Figure 4:**
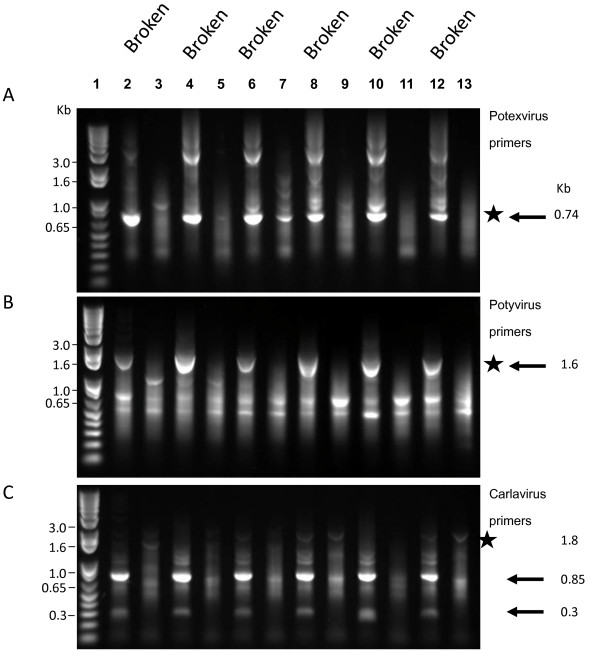
**Amplification of putative viral RNA from tepal extracts of colour-broken and non-colour-broken *Narcissus *flowers**. Thirty cycles of PCR were performed on first strand cDNA using degenerate primers for (A) potexviruses, (B) potyviruses and (C) carlaviruses. The approximate size of the PCR product expected is indicated by a star and arrows indicate the sizes of the amplified products sent for sequencing. Flowers were supplied from two different regions in New Zealand (Nelson, Lanes 4-7 and 12-13; Ashhurst, Lanes 2-3 and 8-11). Lanes 2, 4, 6, 8, 10, 12 show amplification from colour-broken flowers. Lanes 3, 5, 7, 9, 11, 13 from non-colour-broken flowers. Flower cultivars were Lighthouse Reef (lanes 2, 3); Daydream (4, 5); 20/87 seedling (6, 7); Twilight Zone (8, 9); Flying Cloud (10, 11) and Twilight Zone (12, 13). Molecular size markers on left are from 1 Kb Plus DNA Ladder (Invitrogen, Carlsbad, CA).

The association of viral RNA with colour break was examined in a selection of the colour-broken and non-colour-broken flowers used in the indicator plant and TEM analyses (covering two of the geographical regions). Electrophoresis of the putative viral sequences amplified from the tepal cDNA by reverse transcription-polymerase chain reaction (RT-PCR) with degenerate primers to potex-, poty-, and carla-viral family members showed that colour break correlated strongly with the presence of viral RNA (Figure 4A, B, C).

**Figure 5:**
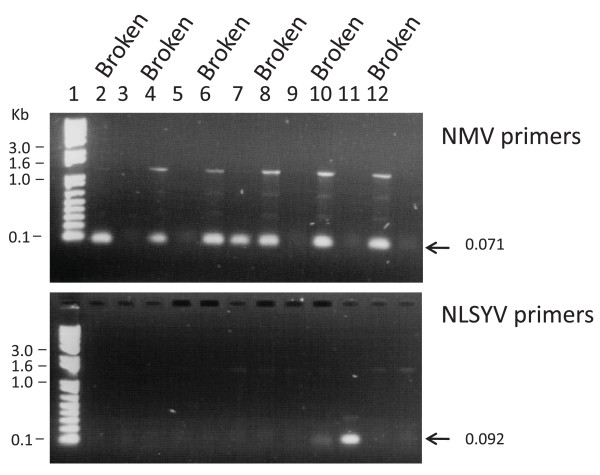
**RT-PCR with NMV- and NLSYV-specific primers on RNA isolated from colour-broken and non-colour-broken *Narcissus *tepals**. Thirty cycles of PCR were performed using primers listed in Table 1. Expected size in bp for the amplified products is indicated. Flowers were supplied from two different regions in New Zealand (Nelson, Lanes 4-7 and 12-13; Ashhurst, Lanes 2-3 and 8-11). Lanes 2, 4, 6, 8, 10, 12 show amplification from colour-broken flowers. Lanes 3, 5, 7, 9, 11, 13 from non-colour-broken flowers. Flower cultivars were Lighthouse Reef (lanes 2, 3); Daydream (4, 5); 20/87 seedling (6, 7); Twilight Zone (8, 9); Flying Cloud (10, 11) and Twilight Zone (12, 13). Molecular size markers on left are from 1 Kb Plus DNA Ladder (Invitrogen).

The potexvirus primers generated a PCR product from the cDNA of colour-broken tepals of ~0.74 kb (Figure 4A), which was the expected size for potexviruses (Table 3). Sequence comparison of the cloned 0.74 kb products amplified from 'Twelve Gauge', 'Daydream' and 'Twilight Zone' colour-broken flowers with sequences in the GenBank indicated that the 0.74 kb fragment from all three flowers showed highest nucleotide similarity (97%) equally to the two NMV accessions in GenBank (New Zealand strain, accession AY225449 and Dutch strain, accession D13747) (Table 3). NMV-specific PCRs performed on the tepal cDNA of the six colour-broken and non-colour-broken flowers (Figure 5), revealed that presence of the potexvirus NMV correlated well with the occurrence of colour break being present in all six colour-broken tepals tested. The potexvirus primers did in one non-colour-broken flower (seedling 20/87, Lane 7 Figure 4A) also produce a 0.74 kb product, but in this instance there was clearly much less amplification which is reflective of much lower (and presumably insufficient) levels of the potexvirus for causing colour break.

**Table 3:** Sequence identification of the viruses present in tepals of *Narcissus *flowers displaying colour break

Cultivar tested	Primer name	Genus expected	Fragment size cloned/expected (kb)	Sequence results	Genus obtained
Twelve Gauge	Potex1RC (For) Potex5(Rev)	Potexvirus	0.74/0.74^1^	NMV (1, 97%)	Potexvirus
	Sprimer (For) KS(Rev)	Potyvirus	1.65/1.60^2^	NLSYV (1, 92%) NMV (6, 97%)	Potyvirus Potexvirus
	pCar-1 (For) KS(Rev)	Carlavirus	0.80/1.80^2^	NMV (2, 98%)	Potexvirus Text
Daydream	Potex1RC (For) Potex5(Rev)	Potexvirus	0.74/0.74^1^	NMV (2, 97%)	Potexvirus
	pCar-1 (For) KS(Rev)	Carlavirus	0.80/1.80^2^ 0.30/1.80^2^	NMV (2, 98%) NMV (2, 98%)	Potexvirus
Twilight Zone	Potex1RC (For) Potex5(Rev)	Potexvirus	0.74/0.74^1^	NMV (1, 97%)	Potexvirus
	Sprimer (For) KS(Rev)	Potyvirus	1.65/1.60^2^	NMV (2, 97%) NLV (4, 98%)	Potexvirus
	pCar-1 (For) KS(Rev)	Carlavirus	0.80/1.80^2^ 0.80/1.80^2^ 0.30/1.80^2^	NSV (1, 95%) NMV (1, 98%) NMV (2, 98%)	Carlavirus Potexvirus
Flying Cloud	Sprimer (For) KS(Rev)	Potyvirus	1.65/1.60^2^	NMV (4, 97%)	Potexvirus

NMV = *Narcissus mosaic virus *(AY225449, D13747); NLSYV = *Narcissus late season yellows virus *(AJ493579); NSV = *Narcissus symptomless virus *(AM182569); NLV = *Narcissus latent virus *(FJ024083). RT-PCR was performed on tepal RNA from four flowers displaying colour break. The predominant amplification products indicated in Figure 4 were gel purified, cloned and sent for sequencing. Numbers in brackets refers to number of clones sequenced and average percentage nucleotide identity to virus accession in GenBank. There are two NMV accessions in the GenBank (New Zealand strain, accession AY225449 and Dutch strain, D13747). Sequences showed equal % similarity to both. Expected size (kb) of PCR product as reported by ^1^van der Vlugt & Berendsen [11], and ^2^Chen et al. [12].

The potyvirus primers generated a PCR product from the cDNA of colour-broken, but not non-colour-broken flowers of ~1.65 kb (Figure 4B), which was close to the expected size for potyviruses (Table 3). However, 12 of the 17 cloned 1.65 kb fragments obtained from either 'Twelve Gauge', 'Flying Cloud' and 'Twilight Zone' colour-broken flowers showed 97% nucleotide similarity to the NMV accessions (AY225449, D13747) and not to potyviruses. This was caused by mis-priming of the S primer to the sequence 5311-CA**A**T**AT**TC**T**T**GTG**A**GCAGCC**-5330 in the 26 K protein of NMV (bases in NMV genomic sequence in common with the primer are in bold and underlined) and binding of the oligo(dT)-based primer (KSDT) to the end of the sequence (position 6956). The potyvirus NLSYV was also identified in one of the sequencing reactions (Table 4). However, NLSYV-specific amplification of this virus revealed that there was no correlation with the presence of this virus and tepal colour break as it was only detected in two of the six colour-broken cultivars tested and was present in both a non-colour-broken and colour-broken flower (Figure 5).

**Table 4:** Primers used in RT-PCR to amplify viral sequences obtained from *Narcissus *flowers

Primer name	Primer sequence
Potex1RC^1 ^(For) Potex5^1 ^(Rev)	5'-TCAGTRTTDGCRTCRAARGT-3' 5'-CAYCARCARGCMAARGAYGA-3'
Sprimer^2 ^(For) KS^3^(Rev)	5'-GGNAAYAAYAGYGGNCARCC-3' 5'-CGGTACCGATAAGCTTGA-3'
pCar-1^2^(For) KS^3^(Rev)	5'-ATGCCNCTNANNCCNCC-3' 5'-CGGTACCGATAAGCTTGA-3'
KSDT	5'-CGGTACCGATAAGCTTGATTTTTTTTTTTTTTTTTTV-3'
NMV_5759_^4 ^(For) NMV_5830_(Rev)	5'-TGCGCTTCTATCTATTGTCTGAGG-3' 5'-GTGGGGAAGGGAATGAATGTTATC-3'
NLSYV_1374_^4 ^(For) NLSYV_1466_(Rev)	5'-TACGACAGAAGAGAACACGGAGAG-3' 5'-ACACAATACTACACGCCCCTTACG-3'

^1^Degenerate primers of van der Vlugt & Berendsen [11], and ^2^Chen et al. [12]; ^3^generic primer that anneals with first strand cDNA synthesis primer, KSDT. N = (A, G, C or T), D = (G+A+T), Y = (T or C), R = (A or G). NMV = *Narcissus mosaic virus*, NLSYV = *Narcissus late season yellows virus*. ^4 ^Primer regions in NMV (accession AY225449) and NLSYV (accession AJ493579). Numbers in subscript identify region in viral genome that primers bind. NMV primers are also perfect match for NMV accession D13747.

The carlaviral primers did not generate an amplification product of expected size for carlaviruses (1.8 kb, Table 3, Figure 4C). Instead, the primers produced products of ca. 0.8 kb and 0.3 kb from cDNA from all six colour-broken flowers but not from the non-colour-broken ones. Nine out of the ten cloned fragments (i.e., five 0.8 kb and four 0.3 kb fragments) from flowers of either 'Twelve Gauge', 'Daydream' and 'Twilight Zone' showed 98% nucleotide similarity to the NMV accessions (AY225449, D13747) (Table 3). The amplification of the 0.8 kb and 0.3 kb NMV sequences was found to be due to primer pCar-1 annealing to the closely related sequences 6218-CC**G**A**CCCTAAGCCCGCC**-6234 and 6698-GCCT**CA**T**TC**G**ACC**T**CCC**-6714 respectively in the coat protein coding region of NMV and the binding of KSDT to the end of the NMV sequence.

## Discussion

Colour break in daffodils can be either a partial phenotype, with mottled colour, or a complete loss of the vivid colour attributed by carotenoids. This lack of uniformity can be a significant problem for commercial production of this important ornamental crop. Therefore, determining whether virus infection is indeed the cause of colour break will help daffodil growers to manage the issue, as well as furthering our understanding of virus interactions with plant metabolic pathways. In this study, a clear association of daffodil flower colour break with viral presence in the perianth has been demonstrated. The association of colour break with virus was confirmed by a number of independent tests such as ability of daffodil tepal sap from colour broken flowers to cause symptoms on indicator plants, direct visualisation of viral particles with TEM, and the ability to amplify viral sequences from the RNA of colour-broken but not non-colour-broken tepals.

The sensitive RT-PCR-based profiling of viral families was a powerful method for determining whether virus (es) were associated with colour break in the reverse bicolour daffodils. To give the best chance of detecting as many different types of viruses as possible, the procedure was carried out at a low annealing temperature with degenerate primers shown previously to amplify successfully members of the potex-, poty- and carlaviral families [11,12]. The clear and consistent differences in the amplification profiles obtained from the six colour-broken flowers compared with the non-colour-broken flowers (Figure 4A, B, C) was striking as the 30 cycles used in the PCR was expected to enable detection of very small quantities of viral RNA. We had initially thought that the profiles would be similar and that the amplification would just be greater in the colour-broken flowers reflecting the presence of higher viral titre in these flowers. However, the inability to amplify products of expected size in most of the non-colour-broken flowers is suggestive of these flowers either not containing or containing extremely low quantities of viruses.

All of the independent tests carried out pointed to NMV being the virus most closely associated with colour break in the reverse bicolour daffodils. For instance, the red ringspot lesions seen in *G. globosa *after inoculation with colour-broken, but not non-colour-broken flowers is characteristic of NMV infection [9], and the TEM observations indicated that the morphology and the lengths of the viral particles in the colour-broken flowers fitted with those reported for NMV [9,13]. However, the most compelling evidence came from sequence analysis and virus-specific RT-PCR profiling. The sequencing of the PCR products amplified from colour-broken flowers with the carla-, poty- and potex-virus degenerate primers all revealed that NMV was the only virus present in all flowers displaying colour break (Table 4). It was particularly telling that the potyviral and carlaviral primers did not predominantly amplify their target family members in PCR, but instead mis-primed to closely related sequences in the NMV genome. The mis-priming to NMV likely occurred because of the absence or very low presence of their respective target family members and the reduced stringency of the PCR achieved by performing it at the low annealing temperature of 47°C. The strong association of NMV with colour break was then able to be confirmed by observing a strong correlation between the occurrence of colour break and the ability to amplify at high stringency (60°C) a PCR product with primers designed specifically to a short region in the NMV genome (Figure 5). This could be contrasted with the poor correlation between the amplification of the potyvirus NLSYV and occurrence of colour break (Figure 5).

NMV was first described and in detail by Brunt [9]. The virus was reported to be widespread in British crops of trumpet, large cupped and double daffodils and NMV was able to be isolated from 27 of the 48 commercial daffodil stocks tested (cultivars 'Actaea', 'Aranjuez', 'Brunswick', 'Carlton', 'Cheerfulness', 'Fortune', 'Golden Harvest', 'Inglescombe', 'King Alfred', 'Magnificence', 'Minister Talma', 'Mount Hood', 'Royal Bride', and 'Zero'). Interestingly, the plants where NMV was the only virus detected were either symptomless or showed inconspicuous mosaic symptoms at the base of their leaves. No mention of colour break was reported for these daffodils, despite their flowers being the best source of NMV particles. However, notably, none of the flowers examined by Brunt [9] were of the reverse bicolour type (J.A. Hunter, Historian of the New Zealand Daffodil Society, *personal communication*).

In a separate study, Clark and Guy [10] surveyed *Narcissus *spp. for viral infection in the Otago Province of New Zealand using ELISA and mechanical transmission tests. A high incidence of viral infection was found in the five sites examined. Indirect ELISA showed that NMV was one of the five viruses detected and that it was present at all five sites in single or mixed infections. They reported that plants infected only with NMV (based on their detection methods) showed mild to conspicuous leaf mosaic symptoms and reported that no colour break symptoms were observed. The absence of colour break among the daffodils surveyed by Clark and Guy [10] is presumably explained by the absence of reverse bicolours amongst the flowers they examined.

Unpublished data by Brunt [14] that "some isolates [of NMV] are associated with flower mottling in cultivars such as Chanter and Spellbinder (A.A. Brunt and S. Phillips, *unpublished information*)" support our findings that NMV infection is closely linked to colour break in reverse bicolours as both of these cultivars are of that type (J.A. Hunter *personal communication*). However, to demonstrate causality will require inoculation experiments. We also cannot rule out that it could be a viral titre phenomenon and not specific to a particular virus.

The ubiquitous occurrence of NMV in daffodil cultivars is intriguing given no vector for transmission has yet been discovered and direct spread by mechanical transmission is slow. Brunt [9] reported it took 17 months after inoculation of daffodil leaves for the virus to be able to be recovered from the leaves. This makes demonstrating Koch's postulate for NMV and colour break in daffodils extremely difficult. First, it would require a set of proven virus-free plants, difficult in itself given the ability of NMV to lie latent at very low titre, secondly, it would require maintaining them in a virus-free environment for a very long period (as the negative controls) and thirdly, it would require having the correct temperature and other conditions for successful virus inoculation.

The widespread occurrence of NMV and the inconspicuous symptoms it produces in many daffodil cultivars makes it difficult for the grower to prevent colour break from occurring as it appears that there is a 'silent' reservoir of the virus which can serve as inocula for infection of reverse bicolours. Because of this, growing this type of flower away from other cultivars may be the best way of preventing their colour break.

## Conclusions

The findings of this study and the previous research have demonstrated that different viruses are responsible for colour break problems in different ornamentals or even different cultivars of the same species. However, little is known about the mechanism of colour break caused by viruses at the cellular level and why in daffodils the reverse-bicolour flowers seem particularly prone to colour break and why NMV seems particularly linked to the process. It is of particular interest that distinct pigment biosynthetic pathways are affected in response to viral infection in tulips (vacuolar-located anthocyanins) and daffodils (plastid-located carotenoids). More study is thus required to understand the molecular basis of viral-induced colour break in flowers.

## Methods

### Plant material

Daffodil material was sourced from three geographic locations in New Zealand; two in the North Island (Ashhurst, and Foxton) and one in the South Island (Nelson). Cut flowers or whole plants were transferred directly to the laboratory from the two nearby locations (Ashhurst and Foxton) and via overnight courier from the distant location (Nelson). Experimental materials were stored either at -20°C or 4°C until needed.

### Viral transmission tests and enzyme-linked immunoassay

Excised daffodil tepal tissues were wrapped in foil and dried in a glass chamber at 4°C in the presence of silica gel and transported overnight in a courier bag from The New Zealand Institute for Plant & Food Research Limited (PFR), Palmerston North site, to the Christchurch PFR site. For viral transmission tests, ~0.1 g tepal tissues were ground in phosphate buffer (pH 7.5) + 0.05% bentonite (modified Yarwood's buffer) and rubbed onto leaves of indicator plants (*Chenopodium amaranticolor*, *Gomphrena globosa*, *Phaseolus vulgaris *and *Nicotiana tabacum*) dusted with carborundum abrasive. The plants were grown at 18-22°C and monitored over 3 weeks for the development of symptoms. Leaf tissues for DIBA-ELISA were crushed and diluted 1:20 in 0.05 M Tris buffer pH 7.5 + 0.2 M NaCl. Duplicate 20 μL drops were spotted on Zeta Probe blotting membrane (Bio-Rad, CA, USA) nitrocellulose paper and processed according to Hammond & Jordan [15] using the broad-spectrum anti-potyviral monoclonal antibody, (AS-0573/1 DSMZ Braunschweig, Germany) at 1:1000 dilution and the goat-anti-mouse alkaline phosphatase conjugate (1:1000) according to manufacturer's instructions.

### Transmission electron microscopy

To observe virus particles, daffodil tepal leaf and bulb tissues were crushed in the same way in a plastic pocket and the slurry then centrifuged at 10000 *x g *for 2 min to clarify the supernatant. TEM grids were then placed on the surface of the droplets of the supernatant. The grids were washed briefly in phosphate-buffered saline (pH 7.2) and negatively stained with 2% uranyl acetate and observed with a Philips 201C TEM. Particle counts were obtained from the photographs taken from at least three randomly chosen fields per TEM grid, and expressed as viral counts per view at magnification of 21200 x.

### Reverse transcriptase polymerase chain reaction and sequencing

RNA was isolated from daffodil tepals that were frozen in liquid nitrogen and ground in a mortar with a pestle using a modified Wan and Wilkins hot borate method [16] described in Hunter *et al. *[17]. First strand cDNA was synthesized using SuperscriptII (Invitrogen, Carlsbad, CA) and an oligo(dT)-based primer KSDT (Table 4). PCR with Qiagen Taq Polymerase (Qiagen, Valencia, CA) was used to amplify the putative viral sequences from the cDNA. The degenerate and virus-specific primers used in PCR to amplify members of the potyvirus, potexvirus and carlavirus genera are listed in Table 4. The composition and utility of the degenerate primers was previously described in [11,12]. The PCR primer KS, complimentary to the 18 bp at the 5' end of the KSDT sequence was used to amplify viral sequences when a suitable reverse viral primer was not known. Conditions for the degenerate PCR consisted of an initial denaturation at 94°C for 5 min followed by 30 cycles of 94°C for 30 s/47°C for 1 min/72°C for 2 min and final extension at 72°C for 5 min.

The primers used for virus-specific PCR to generate short amplicons of NMV and NLSYV were designed using the PrimerSelect module of Lasergene7 software (DNASTAR Inc. Madison, WI, USA) as short amplicons have been shown to increase the sensitivity and accuracy of PCR [18]. The virus-specific primer sequences did not match any non-target virus sequences in the GenBank according to the Basic Local Alignment Search (BLASTN 2.2.18+) algorithm [19] and 'Others' database at http://www.ncbi.nlm.nih.gov. Conditions for the virus-specific PCR consisted of an initial denaturation at 94°C for 5 min followed by 30 cycles of 94°C for 30 s/60°C for 30 s/72°C for 30 s and final extension at 72°C for 5 min. The amplified products were TA-cloned into the pGEM^®^-T Easy vector system II (Promega, Madison, WI). Sequencing was carried out as a commercial service by the DNA Sequencing Facility, University of Waikato, Hamilton, NZ. The identity of the amplified products was confirmed by sequence comparison in GenBank using the Basic Local Alignment Search (BLASTN) algorithm [19] and 'Others' database at http://www.ncbi.nlm.nih.gov.

## Competing interests

The authors declare that they have no competing interests.

## Authors' contributions

DH conceived and organised the project, did the RT-PCR analysis and wrote the manuscript. JF did the indicator plant and DIBA-ELISA analysis. KD contributed to experimental design and writing of the manuscript. HZ contributed to experimental design, did the TEM work and contributed to writing of the manuscript. All the authors have read and approved the final manuscript.
